# ALTA: a simple nutritional prognostic score for patients with hepatitis B virus-related acute-on-chronic liver failure

**DOI:** 10.3389/fnut.2024.1370025

**Published:** 2024-04-09

**Authors:** Rui Song, Xiaohao Wang, Zhao Li, Hongyu Wu, Jiahe Tan, Junyi Tan, Hanlu Li, Teng Zeng, Hong Ren, Zhiwei Chen

**Affiliations:** ^1^Key Laboratory of Molecular Biology for Infectious Diseases, Department of Infectious Diseases, Institute for Viral Hepatitis, The Second Affiliated Hospital of Chongqing Medical University, Chinese Ministry of Education, Chongqing, China; ^2^Department of Gastroenterology, The Seventh People’s Hospital of Chongqing, Chongqing, China; ^3^Department of Hepatobiliary Surgery, The Second Affiliated Hospital of Chongqing Medical University, Chongqing, China; ^4^Department of Neurosurgery, The First Affiliated Hospital of Chongqing Medical University, Chongqing, China; ^5^Department of Infectious Diseases, The Ninth People’s Hospital of Chongqing, Chongqing, China; ^6^Department of Infectious Diseases, The Fifth People’s Hospital of Chongqing, Chongqing, China

**Keywords:** acute-on-chronic liver failure, hepatitis B virus, prognostic score, nutrition, mortality

## Abstract

**Background:**

Malnutrition, despite being a common complication, is often neglected in patients with hepatitis B virus-related acute-on-chronic liver failure (HBV-ACLF). The objective of this study was to develop a simplified nutritional prognostic score to accurately predict mortality in HBV-ACLF patients.

**Methods:**

In this multicenter retrospective study, clinical data from 530 HBV-ACLF patients were used to create a new prognostic score, which was then validated in two external cohorts (*n* = 229 and 248).

**Results:**

Four independent factors were significantly associated with 28-day mortality in HBV-ACLF patients, forming a novel prognostic score (ALTA score = 0.187 × age—0.849 × lymphocyte count—2.033 × total cholesterol—0.148 × albumin—0.971). Notably, the AUROC of ALTA score for 28/90-day mortality (0.950/0.967) were significantly higher than those of three other ACLF prognostic scores (COSSH-ACLF II, 0.864/0.734; MELD, 0.525/0.488; MELD-Na, 0.546/0.517; all *P* < 0.001), and three known nutritional scores (CONUT, 0.739/0.861; OPNI, 0.279/0.157; NRS-2002, 0.322/0.286; all *P* < 0.001). The prediction error rates of ALTA score for 28-day mortality were significantly lower than COSSH-ACLF II (7.3%), MELD (14.4%), MELD-Na (12.7%), CONUT (9.0%), OPNI (30.6%), and NRS2002 (34.1%) scores. Further classifying ALTA score into two strata, the hazard ratios of mortality at 28/90 days were notably increased in the high-risk groups compared to the low-risk group (15.959 and 5.740). These results were then validated in two external cohorts.

**Conclusion:**

ALTA, as a simplified nutritional prognostic score for HBV-ACLF, demonstrates superiority over the COSSH-ACLF II and other scores in predicting short-term mortality among HBV-ACLF patients. Therefore, it may be used to guide clinical management, particularly in primary care settings.

## Introduction

Acute-on-chronic liver failure (ACLF) is a clinical syndrome characterized by an exacerbation of chronic liver disease, associated with a significantly elevated short-term mortality rate (1). The development of early and accurate prognostic indicators, as well as scoring systems, is crucial for optimizing the management of patients presenting with ACLF (2).

To date, numerous prognostic scores have been developed to evaluate the prognosis of ACLF. These scores primarily fall into two categories: the first is based on organ failures (OF), such as the Chronic Liver Failure Consortium Organ Failure (CLIF-C OF) (3), CLIF-C ACLF (4) and the Chinese Group on the Study of Severe Hepatitis B-ACLF (COSSH-ACLF) (2) scores. The second category is derived from liver and kidney function, coagulation, or clinical manifestations, including the Child-Pugh (5), model of end-stage liver disease (MELD) (6), and Asian Pacific Association for the Study of the Liver ACLF research consortium ACLF (AARC-ACLF) (7) scores. Moreover, several modified or simplified scores have been constantly proposed, such as the MELD-Na (8) and COSSH-ACLF II (9) scores. However, most of these scores are not widely applicable, particularly in primary hospitals, due to their complex assessment processes or the inaccuracy of subjective judgments. Consequently, there is an urgent need to develop a simpler, more accessible ACLF prognostic scoring system that utilizes easily obtainable metrics.

Notably, malnutrition is a frequently overlooked yet common complication in advanced liver diseases, which is associated with unfavorable clinical outcomes (10). Some nutritional scoring systems have been employed to assess prognoses in hospitalized patients, such as the Nutritional Risk Screening 2002 (NRS-2002) (11), the Onodera Prognostic Nutritional Index (OPNI) (12), and the Controlling Nutritional Status (CONUT) (13) scores. The primary components of these scoring systems include lymphocyte count, albumin, and total cholesterol level. However, the ability of these nutritional indices to more effectively identify ACLF patients with poor prognoses remains unknown.

Given that hepatitis B virus (HBV) infection is the primary etiology of ACLF in China (14), hence, the current study aims to develop a new simple nutritional prognostic score to accurately predict short-term mortality in patients with HBV-ACLF.

## Patients and methods

### Study design

Retrospectively enrolling patients with HBV-ACLF from January 2021 to June 2023 at the Second Affiliated Hospital of Chongqing Medical University, we utilized a two-step approach to develop a novel prognostic score for HBV-ACLF patients. Firstly, clinical data and selected nutritional indices from these patients were employed to identify predictive factors associated with short-term (28-day and 90-day) mortality, laying the foundation for the construction of the new prognostic score. Secondly, a comprehensive external validation was conducted using two independent cohorts of HBV-ACLF patients from the Fifth People’s Hospital of Chongqing (January 2015 to June 2023) and the Ninth People’s Hospital of Chongqing (January 2018 to June 2023). This study was approved by the Ethics Committee of the Second Affiliated Hospital of Chongqing Medical University and two other hospitals (Ratification No. 87/2023), and adhered to the ethical guidelines stipulated by the Declaration of Helsinki. Due to the retrospective nature of the study, patient informed consent was waived by the Committees.

### Patients

The inclusion criteria were as follows: (i) aged 18 years or older; (ii) positivity of serum hepatitis B surface antigen for at least 6 months; (iii) fulfilling the ACLF criteria of APASL (15) {previously diagnosed or undiagnosed chronic liver disease/cirrhosis, characterized by jaundice [serum total bilirubin (TBil) ≥ 5 mg/dl] and coagulation disorder [international normalized ratio (INR) ≥ 1.5 or prothrombin activity (PTA) < 40%], with concurrent ascites and/or hepatic encephalopathy (HE) within 4 weeks}; (iv) availability of baseline data for lymphocyte count, total cholesterol, and albumin. Exclusion criteria: (i) presence of acute (an illness duration of < 26 weeks duration in a patient without preexisting liver disease or cirrhosis associated with any degree of HE and INR ≥ 1.5) (16) or chronic liver failure (a slow progressive decline and decompensation of liver function on the basis of cirrhosis) (17); (ii) chronic liver diseases of other etiology; (iii) hepatic or extra-hepatic malignancies; (iv) use of immunosuppressants or corticosteroids; (v) incomplete clinical data. All patients underwent standard medical treatments, including antiviral agents for HBV-DNA positive patients, management for ascites, HE, and/or bacterial infections. Artificial liver support was administered after comprehensive evaluation and informed consent. Clinical data were collected at admission, encompassing demographic information, medical history, clinical parameters, and laboratory indicators. The 28-day and 90-day liver transplant-free survival rates of each patient were also assessed.

### Scoring models

The MELD score (6) was calculated using the following formula: MELD = 3.78 × ln[TBil, (mg/dL)] + 11.2 × ln(INR) + 9.57 × ln[serum creatinine (mg/dL)] + 6.43.

The MELD-Na score (8) was derived from the MELD score using the formula: MELD-Na = MELD—Na—[0.025 × MELD × (140—Na)] + 140, with the serum sodium concentration ranging between 125 and 140 mmol/L.

The COSSH-ACLF II score (9) was calculated based on the formula: COSSH-ACLF II = 1.649 × ln(INR) + 0.457 × HE score + 0.425 × ln(neutrophil) + 0.396 × ln(TBil) + 0.576 × ln(serum urea) + 0.033 × age.

The NRS-2002 score (11) was assessed through three components: impaired nutritional status, disease severity, and age ≥ 70 years, with a score of ≥ 3 indicating nutritional risk.

The OPNI score (12) was calculated as 10 × albumin (g/L) + 5 × lymphocyte count (10^9^/L).

The CONUT score (13) was derived from the sum of the albumin score, lymphocyte count score, and total cholesterol score.

All scores were applied at the time of admission for each patient in this study. Due to the arterial blood gas analysis not being performed in most ACLF patients at admission, the scores related to SOFA or lactate level could not be evaluated in this study.

### Statistical analysis

Appropriate statistical methods were employed based on the type of data, with categorical variables represented as percentages (%), and continuous variables reported as median with interquartile range (IQR). Comparisons between categorical variables were conducted using the Chi-Square test or Fisher’s exact test, while the Mann–Whitney U test was applied for continuous variables. Logistic regression analysis was utilized to identify the independent prognostic predictors of 28-day mortality. Variance inflation factors (VIFs) were calculated to assess collinearity, and variables with a VIF exceeding 10 were excluded. Variables with a *P* < 0.1 value in the univariate logistic regression analysis were advanced to a multivariate analysis using stepwise regressions. Receiver operator characteristic (ROC) curves were generated for predictors, and the area under ROC curve (AUROC) was employed to evaluate their predictive value. AUROCs were compared using the DeLong method. The probability density function (PDF) was utilized to define an integral of the survival and non-survival densities within a specified range of each score, while the difference in overlapping coefficient between ALTA and other scores was assessed using the Chi-Square test. The optimal cut-off value of the new prognostic score was determined based on selecting the largest v2 value using X-tile software (18) (version 3.6.1) to categorize patients into low-risk and high-risk groups for mortality. Hazard ratios (HRs) of 28-day and 90-day mortality among the three groups were calculated by Cox regression analysis. Cumulative survival rates were compared using the Kaplan-Meier method. Decision curve analysis (DCA) was employed to assess the new prognostic score in clinical practice by examining the theoretical relationship between the threshold probability of an event occurring and the relative value of false-positive and false-negative results. This method could determine if predictive models are clinically effective (19). A two-sided *p*-value < 0.05 was considered statistically significant. Statistical analysis was performed using SPSS (version 24.0.0), and graphs were created using GraphPad Prism (version 8.0.2) and R (version 4.3.2).

## Results

### Characteristics of patients with HBV-ACLF

In this study, a total of 967 ACLF patients were recruited. Following screening, 530 eligible HBV-ACLF patients were selected for the ultimate analysis (Supplementary Figure 1). Table 1 summarizes the clinical features of HBV-ACLF patients at the time of admission. Of the 530 patients, the median age was 65.0 years, with males comprising over 50% (68.1%). Most patients presented with cirrhosis (65.7%), followed by ascites (only), HE (only) and ascites +HE (43.0, 33.2 and 17.4%, respectively). Regarding hepatitis B, 36.4% of HBV-ACLF patients were HBeAg-positive, while 79.8% were receiving antiviral therapy [entecavir (47.3%), tenofovir (47.5%), and entecavir+tenofovir (5.2%)]. According to the CONUT score, all patients exhibited varying degrees of malnutrition, i.e., 56 (10.1%), 275 (51.9%), and 202 (39.1%) patients had mild, moderate, and severe malnutrition, respectively. Among the 530 HBV-ACLF patients, no liver transplantation was performed, and the mortality rates at 28/90 days were 40.6 and 61.7%, respectively. As depicted in Table 1, 28-day survivors tended to be younger, slighter malnutrition, and exhibit higher lymphocyte count, albumin, and total cholesterol compared to non-survivors. A similar trend was also observed in the 90-day analysis (Table 1).

**TABLE 1:** Baseline characteristics of patients with HBV-ACLF in the derivation cohort.

Characteristics	Total (*n* = 530)	28-day survivor (*n* = 315)	28-day non-survivor (*n* = 215)	*p*-value	90-day survivor (*n* = 203)	90-day non-survivor (*n* = 327)	*p*-value
Age, (years)	65.0 (47.0–65.0)	49.0 (46.5–55.0)	67.0 (55.5–71.0)	< 0.001	47.0 (45.0–53.0)	56.0 (53.0–69.0)	< 0.001
Gender, (male)	361 (68.1)	217 (68.9)	144 (67.0)	0.704	152 (74.9)	209 (63.9)	0.032
Current smoking	258 (48.7)	144 (45.7)	114 (53.0)	0.111	103 (50.7)	155 (47.4)	0.475
Current drinking	187 (35.3)	115 (36.5)	72 (38.6)	0.517	71 (35.0)	116 (35.5)	0.926
HBeAg positive	193 (36.4)	110 (34.9)	83 (34.9)	0.409	64 (31.5)	129 (39.4)	0.078
Cirrhosis	348 (65.7)	207 (65.7)	141 (65.6)	1.000	117 (57.6)	231 (70.6)	0.003
Ascites (only)	228 (43.0)	106 (33.7)	122 (56.7)	0.185	33 (16.3)	195 (59.6)	< 0.001
HE (only)	176 (33.2)	81 (27.0)	95 (44.2)	< 0.001	58 (28.6)	118 (36.1)	0.074
Ascites+HE	92 (17.4)	25 (7.9)	67 (31.1)	< 0.001	12 (6.0)	80 (24.5)	< 0.001
Antiviral history				0.179			0.911
Absent	107 (20.2)	60 (19.0)	47 (21.9)		40 (19.7)	67 (20.5)	
Present	423 (79.8)	255 (81.0)	168 (78.1)		163 (80.3)	260 (79.5)	
Antiviral agents using				0.353			0.444
Entecavir	200 (47.3)	136 (48.6)	62 (43.4)		79 (48.5)	121 (46.5)	
Tenofovir	201 (47.5)	130 (46.4)	71 (49.7)		77 (47.2)	124 (47.7)	
Entecavir+tenofovir	22 (5.2)	12 (4.3)	10 (7.0)		7 (4.3)	15 (5.8)	
Laboratory examination							
RBC (10^12^/L)	3.3 (2.6–3.9)	3.3 (2.6–4.1)	3.3 (2.8–3.8)	0.891	3.3 (2.6–4.1)	3.3 (2.7–3.8)	0.944
WBC (10^9^/L)	5.7 (4.3–8.4)	5.4 (4.2–8.9)	5.9 (4.7–7.7)	0.704	4.6 (4.0–9.2)	6.6 (4.8–8.4)	0.001
Lymphocyte (10^9^/L)	0.9 (0.5–1.4)	1.3 (0.7–1.8)	0.5 (0.4–0.9)	< 0.001	1.4 (1.2–2.2)	0.5 (0.3–0.9)	< 0.001
Neutrophil (10^9^/L)	6.3 (3.2–8.9)	6.4 (3.5–9.0)	6.1 (2.8–8.7)	0.062	6.4 (3.8–9.7)	6.1 (3.2–8.7)	0.166
Platelet (10^9^/L)	83.5 (51.3–114.0)	74.0 (55.5–114.0)	84.0 (48.0–111.0)	0.300	60.0 (53.0–111.0)	84.0 (51.0–114.0)	0.001
Albumin (g/L)	30.0 (27.1–32.5)	30.1 (27.5–36.0)	29.0 (25.8–32.4)	< 0.001	31.2 (29.9–36.6)	29.0 (25.0–32.3)	< 0.001
ALT (U/L)	111.0 (42.5–285.0)	86.0 (27.0–188.0)	129.0 (58.0–625.0)	< 0.001	65.0 (19.0–188.0)	115.0 (56.0–583.0)	< 0.001
TB (mg/dL)	11.4 (5.1–16.3)	12.0 (5.1–17.4)	11.4 (5.2–15.6)	0.811	7.6 (4.9–13.6)	12.7 (5.2–17.4)	0.001
Serum creatinine (μmol/L)	60.8 (48.0–73.9)	62.6 (51.40–72.9)	54.9 (47.4–76.6)	0.001	62.0 (51.4–73.0)	57.5 (45.9–73.9)	0.013
BUN (mmol/L)	6.6 (4.1–10.3)	6.7 (4.4–10.3)	6.5 (3.8–9.5)	0.289	6.4 (4.0–10.3)	6.8 (4.2–10.3)	0.747
Serum sodium (mmol/L)	133.1 (125.0–140.9)	133.3 (125.1–140.2)	132.9 (124.9–142.6)	0.735	133.9 (124.8–140.9)	132.9 (125.1–132.9)	0.656
PT	22.7 (19.1–24.6)	21.5 (18.2–24.0)	23.2 (21.5–26.2)	< 0.001	20.8 (18.0–23.3)	24.0 (21.9–26.1)	< 0.001
INR	1.7 (1.1–1.9)	1.5 (1.0–1.6)	1.7 (1.0–1.9)	< 0.001	1.4 (1.0–1.4)	1.8 (1.0–1.9)	< 0.001
TC (mmol/L)	2.3 (1.8–3.0)	2.7 (2.2–3.8)	1.8 (1.6–2.0)	< 0.001	3.0 (2.6–4.2)	1.9 (1.6–2.5)	< 0.001
Nutrition index^#^				< 0.001			< 0.001
Normal	0	0	0		0	0	
Mild malnutrition	56 (10.1)	56 (17.8)	0		50 (24.6)	3 (0.9)	
Moderate malnutrition	275 (51.9)	182 (57.8)	93 (43.3)		152 (74.9)	123 (37.6)	
Severe malnutrition	202 (39.1)	77 (24.4)	122 (56.7)		1 (0.5)	201 (61.5)	

Data are presented in *n* (%) or median (IQR) as appropriate. ^#^The nutrition status was classified by the CONUT scoring system. ALT, alanine aminotransferase; BUN, blood urea nitrogen; HE, hepatic encephalopathy; IQR, interquartile range; PT, prothrombin time; RBC, red blood cell; TB, total bilirubin; TC, total cholesterol; WBC, white blood cell.

### Development of a new prognostic score

To develop a simplified, practicable nutritional prognostic score for patients with HBV-ACLF, we collected clinical and laboratory indices at admission to identify the most significant risk factors associated with 28-day mortality. After excluding collinearity variables using VIF (Supplementary Table 1), univariate analysis revealed that age, HE, lymphocyte count, albumin, TBil, and total cholesterol were significantly associated with 28-day prognosis in patients with ACLF (Supplementary Table 2). But antiviral history was not associated with short-term mortality. Finally, multivariate analysis identified four independent risk factors for the final prognostic score: albumin, lymphocyte count, total cholesterol, and age (Supplementary Table 2). Based on the multivariate logistic regression analysis, the novel prognostic model for patients with HBV-ACLF (ALTA score) was calculated using the following formula: ALTA score = 0.187 × age (years)—0.849 × lymphocyte count (10^9^/L)—2.033 × total cholesterol (mmol/L)—0.148 × albumin (g/L)—0.971.

### Evaluation of the new score

**FIGURE 1:**
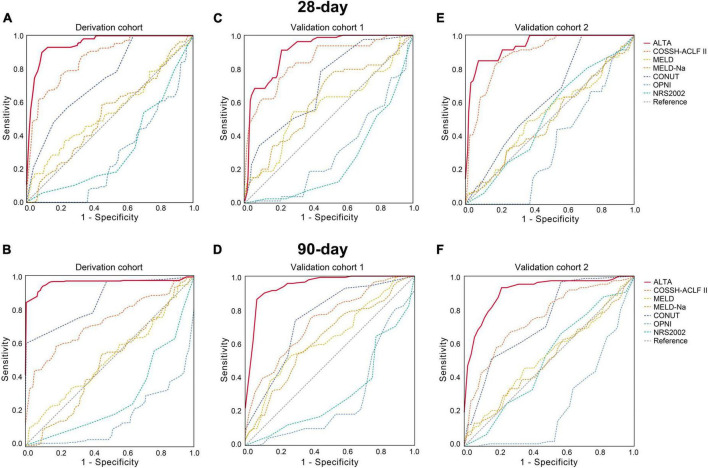
AUROC of the ALTA score for predicting mortality of HBV-ACLF patients in the derivation and validation cohorts. The AUROC of the ALTA score for predicting 28-day and 90-day mortality of patients in the derivation cohort **(A,B)**, the validation cohort 1 **(C,D)**, and the validation cohort 2 **(E,F)**.

**FIGURE 2:**
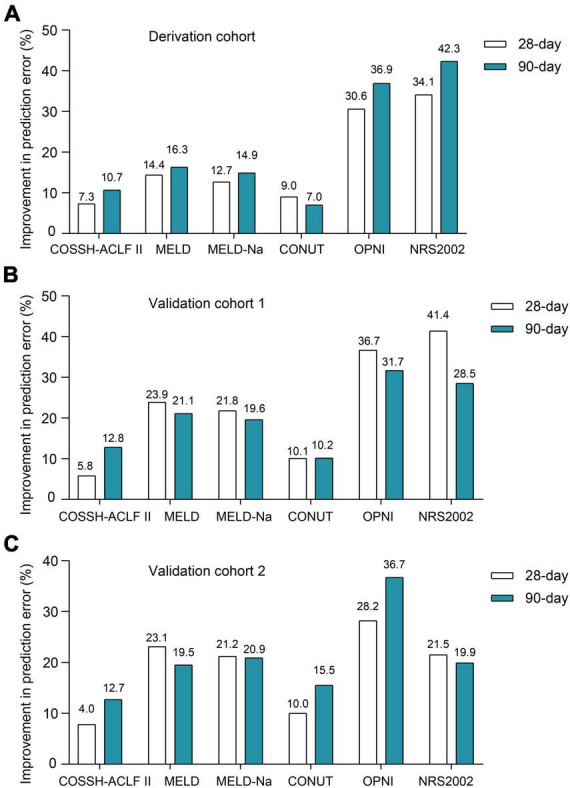
The improvement prediction error rates for 28-/90-day mortality of HBV-ACLF patients for the ALTA score. Compared to other scores, percent reduction in the prediction error rates for 28-/90-day mortality of the ALTA score in the derivation cohort **(A)**, the validation cohort 1 **(B)** and the validation cohort 2 **(C)**.

First, we evaluated the discriminative ability of the new ALTA score. As shown in Figure 1, the area under the receiver operating characteristic curve (AUROC) for 28-day and 90-day mortality of the ALTA score demonstrated a higher predictive accuracy (0.950 and 0.967, respectively), compared to the three other ACLF prognostic scores (COSSH-ACLF II: 0.845 and 0.814, MELD: 0.521 and 0.519, MELD-Na: 0.606 and 0.571; all *P* < 0.001), as well as three established nutritional scores (CONUT: 0.739 and 0.861, OPNI: 0.279 and 0.157, and NRS-2002: 0.322 and 0.286; all *P* < 0.001; Table 2). Furthermore, ALTA demonstrated the highest prognostic accuracy for 28-day mortality compared to COSSH-ACLF II (7.3%), MELD (14.4%), MELD-Na (12.7%), CONUT (9.0%), OPNI (30.6%), and NRS2002 (34.1%), as evidenced by the corresponding percent improvement in prediction error rates obtained with ALTA when compared to other scores (Figure 2A). The improvement in prediction error rates for 90-day mortality was more obvious by ALTA. Further subgroup analysis in cirrhosis revealing that the ALTA score exhibits robust predictive performance across both patients with and without cirrhosis (Supplementary Figure 3 and Supplementary Table 4).

**TABLE 2:** The AUROC of ALTA for predicting 28-/90-day mortality of patients with HBV-ACLF.

Model	ALTA	COSSH-ACLF II	MELD	MELD-Na	CONUT	OPNI	NRS2002
**Derivation cohort**							
28-day mortality	0.950	0.864	0.525	0.546	0.739	0.279	0.322
*p*-value		< 0.001	< 0.001	< 0.001	< 0.001	< 0.001	< 0.001
90-day mortality	0.967	0.734	0.488	0.517	0.861	0.157	0.286
*p*-value		< 0.001	< 0.001	< 0.001	< 0.001	< 0.001	< 0.001
**Validation cohort 1**							
28-day mortality	0.909	0.848	0.573	0.616	0.705	0.301	0.248
*p*-value		< 0.001	< 0.001	< 0.001	< 0.001	< 0.001	< 0.001
90-day mortality	0.950	0.733	0.624	0.633	0.742	0.277	0.307
*p*-value		< 0.001	< 0.001	< 0.001	< 0.001	< 0.001	< 0.001
**Validation cohort 2**							
28-day mortality	0.941	0.888	0.502	0.525	0.632	0.345	0.519
*p*-value		< 0.001	< 0.001	< 0.001	< 0.001	< 0.001	< 0.001
90-day mortality	0.913	0.751	0.512	0.529	0.735	0.248	0.510
*p*-value		< 0.001	< 0.001	< 0.001	< 0.001	< 0.001	< 0.001

**FIGURE 3:**
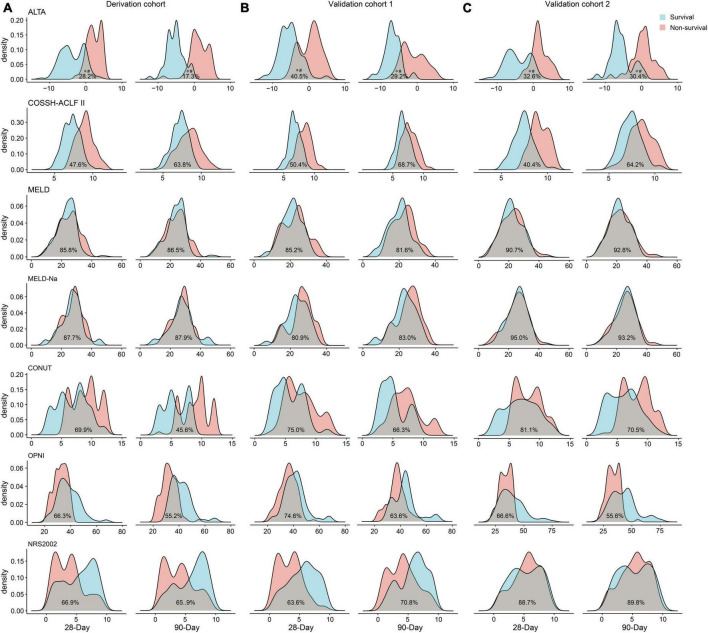
Probability density function of the ALTA score for the 28-/90-day prognosis of surviving and non-surviving patients. The derivation cohort **(A)**, the validation cohort 1 **(B)**, and the validation cohort 2 **(C)**. *p < 0.001 for comparisons of the overlapping coefficient between ALTA score and the other scores. ^#^*p* < 0.001 for comparisons of the ALTA score between surviving and non-surviving patients.

The results of PDF analysis revealed a positive correlation between increasing ALTA score and the proportion of patients with poor outcomes, with distinct peaks observed for surviving and non-surviving patients (Figure 3A). Notably, PDF analysis demonstrated significantly lower overlapping coefficients for ALTA in relation to 28-/90-day mortality (28.2%/17.3%) compared to COSSH-ACLF II (47.6%/63.8%), MELD (85.8%/86.5%), MELD-Na (87.7%/87.9%), CONUT (69.9%/45.6%), OPNI (66.3%/55.2%), and NRS2002 (66.9%/65.9%) scores (all *P* < 0.001), indicating superior prognostic accuracy of the new ALTA score through reduced similarity between probability distributions for surviving and non-surviving patients.

**FIGURE 4:**
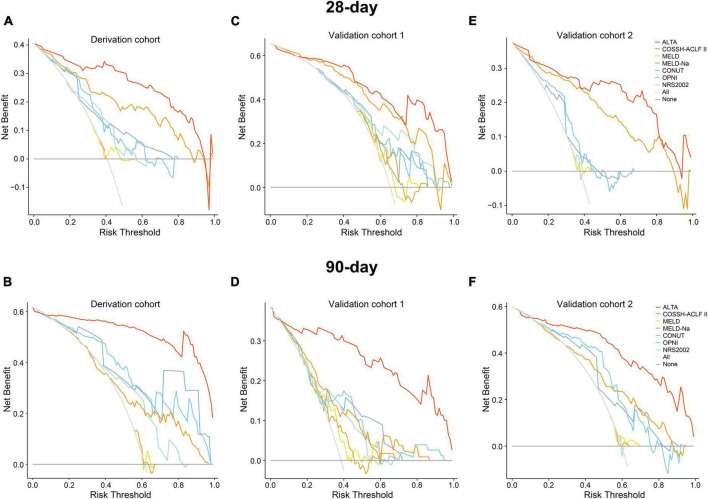
Decision curve analysis of the ALTA score for predicting mortality of HBV-ACLF patients in the derivation and validation cohorts. The decision curve analysis of the ALTA score for predicting 28-day and 90-day mortality of patients in the derivation cohort **(A,B)**, the validation cohort 1 **(C,D)**, and the validation cohort 2 **(E,F)**.

Last, we assessed the clinical utility of the ALTA score. The decision curve analysis (DCA) demonstrated a substantial net benefit for the ALTA score across a broad range of threshold probabilities in predicting 28-day and 90-day mortality, which was larger than other scores (Figure 4).

Taken together, the ALTA score demonstrates superior discriminative ability, accuracy, and enhances clinical decision-making compared to other prognostic scores.

### Risk stratification of the new score

**FIGURE 5:**
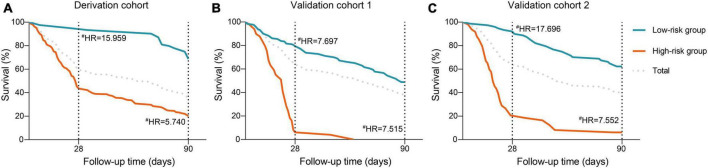
Risk stratification of the ALTA score. Cumulative incidence of mortality at 28- and 90-day stratified according to the ALTA classification rule (low-/high-risk: ALTA score ≤−0.2/>−0.2) in the in the derivation cohort **(A)**, the validation cohort 1 **(B)**, and the validation cohort 2 **(C)**, ^#^*P* < 0.001 for hazard ratios of death in the high-risk groups compared with those in the low-risk group. HR, hazard ratio.

Subsequently, we interrogate whether the novel scoring system has the capacity to effectively stratify the short-term mortality of HBV-ACLF patients. As depicted in the X-tile plot (Supplementary Figure 2), HBV-ACLF patients were hierarchically categorized into two risk strata for death within 28 days, based on one optimal cut-off values (−0.2): low-risk (≤ −0.2) and high-risk (> −0.2). The 28/90-day mortality rates for each group exhibited a pronounced discrepancy (low-risk, 4.9%/25.2%; high-risk, 56.4%/77.9%; Figure 5). In comparison to the low-risk group, the hazard ratios (HRs) for mortality at 28/90 days were notably elevated in the high-risk groups (15.96/5.74, all *P* < 0.001; Figure 5).

### External validation of the new score

Patients with HBV-ACLF from two other hospitals (*n* = 229 and 248) were utilized as two external cohorts to validate the novel scoring system (Supplementary Table 3). The ALTA score outperformed all other scores such as COSSH-ACLF II, MELD, MELD-Na, CONUT, OPNI, and NRS-2002 (all *P* < 0.01) in terms of AUROC (Figure 1 and Table 2). The new ALTA score showed a significant improvement in the prediction errors for 28-/90-day mortality compared to other scores (4.0 to 41.4%; Figures 2B, C).

The PDF analysis also revealed a reduced overlapping coefficient of the ALTA score between the surviving and non-surviving patients in the validation cohorts (all *P* < 0.001; Figures 3B, C). In addition, the DCA cure analysis demonstrated that the ALTA score possesses the largest net benefit for predicting 28/90-day mortality than other scores in validation cohorts (Figure 4). The HRs of death at 28/90-day in the high-risk groups of the validation cohorts resembled those in the derivation group compared to the low-risk group (validation cohort 1: 7.70/7.52, all *P* < 0.001; validation cohort 2: 17.70/7.55, all *P* < 0.001; Figure 5).

In brief, our findings indicate an enhancement in the predictive efficacy of the novel ALTA score for short-term (28/90-day) mortality, as compared to other generic prognostic scores and nutritional assessment tools.

## Discussion

Malnutrition, an important yet frequently overlooked factor, significantly impacts the prognosis of patients with end-stage liver disease, particularly those with ACLF (10). Even though numerous prognostic scores have been developed for ACLF patients, those related to nutrition remain scarce. In this multicenter retrospective study, we concentrated on the nutrient status of patients with HBV-ACLF and their short-term prognosis, and accordingly, developed a novel, simple, and user-friendly score, the ALTA, which accurately predicts the 28/90-day mortality of HBV-ACLF patients.

Early prognosis determination in ACLF patients can guide clinical management to reduce short-term mortality (2). Consequently, numerous new scoring models have been proposed to better achieve this goal. The MELD score, initially developed to evaluate cirrhosis patients’ prognosis after TIPS, has been used for organ allocation (6). However, several studies have shown that the MELD score and its derivative (MELD-Na) have a relatively low accuracy in predicting short-term mortality in ACLF patients (20–22), consistent with our observations. Additionally, organ failure-based scores like the CLIF-ACLF score (4), COSSH-ACLF score (2) and AARC-ACLF score (7), have demonstrated good prognostic abilities in ACLF patients. However, these scores cannot be widely used in primary hospitals due to complexity in organ failure scoring or lack of arterial blood gas data. Therefore, it’s crucial to develop a simplified prognostic score for ACLF patients.

The recently proposed COSSH-ACLF II score is a simplified version of the COSSH-ACLF score, containing six independent risk factors reflecting HBV-ACLF pathophysiology. This score has better predictive power in predicting the prognosis of HBV-ACLF patients compared to the COSSH-ACLF and CLIF-C ACLF scores (9). However, it doesn’t consider HBV-ACLF patients’ nutrition status. In this study, we developed a simple, nutrition-based ALTA score for HBV-ACLF patients, consisting of four indices (albumin, lymphocyte count, total cholesterol, and age). Notably, the ALTA score outperformed the COSSH-ACLF II score in predicting 28/90-day mortality in HBV-ACLF patients, which showed in higher AUROC value and DCA net benefit, and lower prediction error rate for 28/90-day mortality and overlapping coefficient in distributions for surviving and non-surviving patients. However, arterial blood gases at admission were not determined in most ACLF patients, hindering direct comparison of these scores. The ALTA score might be superior to these organ failure-based scores, based on the comparison results of the COSSH-ACLF II scores.

The NRS-2002 score, a widely used nutritional screening tool for inpatients (11), demonstrated low predictive ability for short-term mortality in HBV-ACLF patients in our study. This suggests that general nutritional scores might not be suitable for HBV-ACLF patients. The OPNI score, a simple prognostic tool for preoperative nutritional status (12), and the CONUT score, another nutritional risk screening tool (13), both showed limited predictive value in HBV-ACLF patients. Similarly, in this study, OPNI and CONUT scores showed low and intermediate abilities in predicting the mortality of HBV-ACLF patients, respectively. Therefore, it’s crucial to develop a more accurate nutritional prognostic score tailored to HBV-ACLF patients. Surprisingly, the new ALTA score, which only added age factor compared to the CONUT score (12), exhibited significantly higher predictive ability for HBV-ACLF patients compared to the other three widely used nutritional scores.

The ALTA score, differing from previous prognostic scores based on ACLF pathophysiology, primarily focused on the nutritional status of patients with HBV-ACLF. Low serum albumin levels may result in specific antioxidant function or systemic protein metabolism and inflammation abnormalities (23, 24), which can lead to an increase in infections, renal dysfunction, refractory ascites, and accelerate the progress of ACLF and mortality rates (25, 26). Decreased lymphocyte count, reflecting reduced immune and inflammatory status (27), which may exacerbate the immunosuppression in ACLF (28). The lymphocyte count, especially for CD8^+^ T cell, have been found significantly decreased in non-survivors compared with survivors of HBV-ACLF (29). Similarly, studies suggest that decreased total cholesterol represents deterioration of nutritional status and exacerbates inflammation, posing a higher risk of death in older patients (30, 31). Interestingly, a previous study found hypercholesterolemia was associated with well-preserved hepatic function and decreased mortality in patients with cirrhosis (32). On the other hand, a recent study showed that HBV-ACLF patients with lower high-density lipoprotein cholesterol level had a worse prognosis than those with higher HDL-C levels (33). Increased age also raises the risk of mortality due to a higher incidence of comorbidities and poor hepatic regeneration in response to acute insults (34). These mechanisms, at least in part, explain the accuracy of the ALTA score in predicting the prognosis of patients with HBV-ACLF. As the ACLF pathophysiology involves no unique factors, the ALTA score (or an optimized version) may prove universal for other ACLF etiologies or end-stage liver diseases (e.g., hepatocellular carcinoma), and even for patients with malignant tumors. Further studies to validate this hypothesis are greatly appreciated.

Cirrhosis is closely associated with the development of ACLF. The impact of cirrhosis on mortality in ACLF patients remains controversial, previous study suggesting that cirrhosis independently predicts short-term mortality in ACLF patients (35), while others argue that it is not an independent risk factor for ACLF-related death (36). In our study, we observed no significant correlation between the presence of cirrhosis and the risk of death at 28 or 90 days. Additionally, we evaluated ALTA score comprising both cirrhotic and non-cirrhotic patients, demonstrating their effective predictive performance in both cohorts, which hint that the ALTA score may be widely applicable for patients with ACLF. In addition, whether antiviral therapy is associated with HBV-ACLF survival remains controversial (37, 38). In this study, antiviral therapy was not an independent risk factor of HBV-ACLF short-term survival. More studies with large sample size are highly appreciated to verify our results.

To our knowledge, the ALTA score is the first nutrition-based prognostic score specific to HBV-ACLF patients, accurately predicting short-term mortality. However, the study has limitations. Firstly, despite being a multicenter study, the retrospective nature inherently lead to an unavoidable selection bias. And most patients were recruited from Chongqing in this study, which cannot represent other region of China and the world. Large-sample, prospective, and national studies are needed. Additionally, arterial blood gases at admission were not determined in most patients, hindering direct comparison with organ failure-based scores. However, indirect comparison was made through the COSSH-ACLF II study. Lastly, we cannot evaluate the effect of early intervention for high-risk HBV-ACLF patients identified by the ALTA score. A well-designed prospective study is highly appreciated.

## Conclusion

In summary, we have successfully devised the ALTA score, a concise and accurate predictive tool capable of stratifying the short-term mortality risk of HBV-ACLF patients. This score, which employs only four readily accessible predictor factors, could holds promise for guiding the management of patients diagnosed with HBV-ACLF, particularly for clinicians in primary hospitals. However, further validation and assessment of the ALTA score’s clinical utility are necessary, which may be achieved through larger, prospective studies in the future.

## Data availability statement

The raw data supporting the conclusions of this article will be made available by the authors, without undue reservation.

## Ethics statement

The studies involving humans were approved by the Ethics Committee of the Second Affiliated Hospital of Chongqing Medical University. The studies were conducted in accordance with the local legislation and institutional requirements. The Ethics Committee/Institutional Review Board waived the requirement of written informed consent for participation from the participants or the participants’ legal guardians/next of kin because due to the retrospective nature of the study, patient informed consent was waived by the Committees.

## Author contributions

RS: Conceptualization, Data curation, Formal analysis, Funding acquisition, Investigation, Methodology, Project administration, Resources, Software, Supervision, Validation, Visualization, Writing – original draft, Writing – review & editing. XW: Conceptualization, Data curation, Formal analysis, Funding acquisition, Investigation, Methodology, Project administration, Resources, Supervision, Validation, Visualization, Writing – review & editing. ZL: Conceptualization, Investigation, Methodology, Project administration, Resources, Supervision, Validation, Visualization, Writing – review & editing. HW: Data curation, Formal analysis, Methodology, Project administration, Software, Writing – review & editing. JiT: Data curation, Formal analysis, Methodology, Software, Supervision, Validation, Writing – review & editing. JuT: Data curation, Investigation, Validation, Writing – review & editing. HL: Data curation, Formal analysis, Software, Validation, Writing – review & editing. TZ: Conceptualization, Data curation, Software, Writing – review & editing. HR: Resources, Validation, Visualization, Writing – original draft, Writing – review & editing. ZC: Conceptualization, Methodology, Visualization, Writing – original draft, Writing – review & editing.
