# Supplementary material for: ALTA: a simple nutritional prognostic score for patients with hepatitis B virus-related acute-on-chronic liver failure
Source: Front Nutr. 2024 Apr 9;11:1370025. doi: 10.3389/fnut.2024.1370025 (PMC11035766; doi:10.3389/fnut.2024.1370025)
Supplement: Supplementary file 1 [file Data_Sheet_1.docx]

**ALTA: a simple nutritional** **prognostic score for patients with hepatitis B virus-related acute-on-chronic liver failure**

Rui Song, Xiaohao Wang, Zhao Li, Jiahe Tan, Hongyu Wu, Junyi Tan, Hanlu Li, Teng Zeng, Hong Ren, Zhiwei Chen

**Table of contents**

**Figure S1............................................................................................................2**

**Figure S2............................................................................................................3**

**Figure S3............................................................................................................4**

**Table S1.............................................................................................................5**

**Table S2..............................................................................................................6**

**Table S3..............................................................................................................7**

**Table S4..............................................................................................................8**


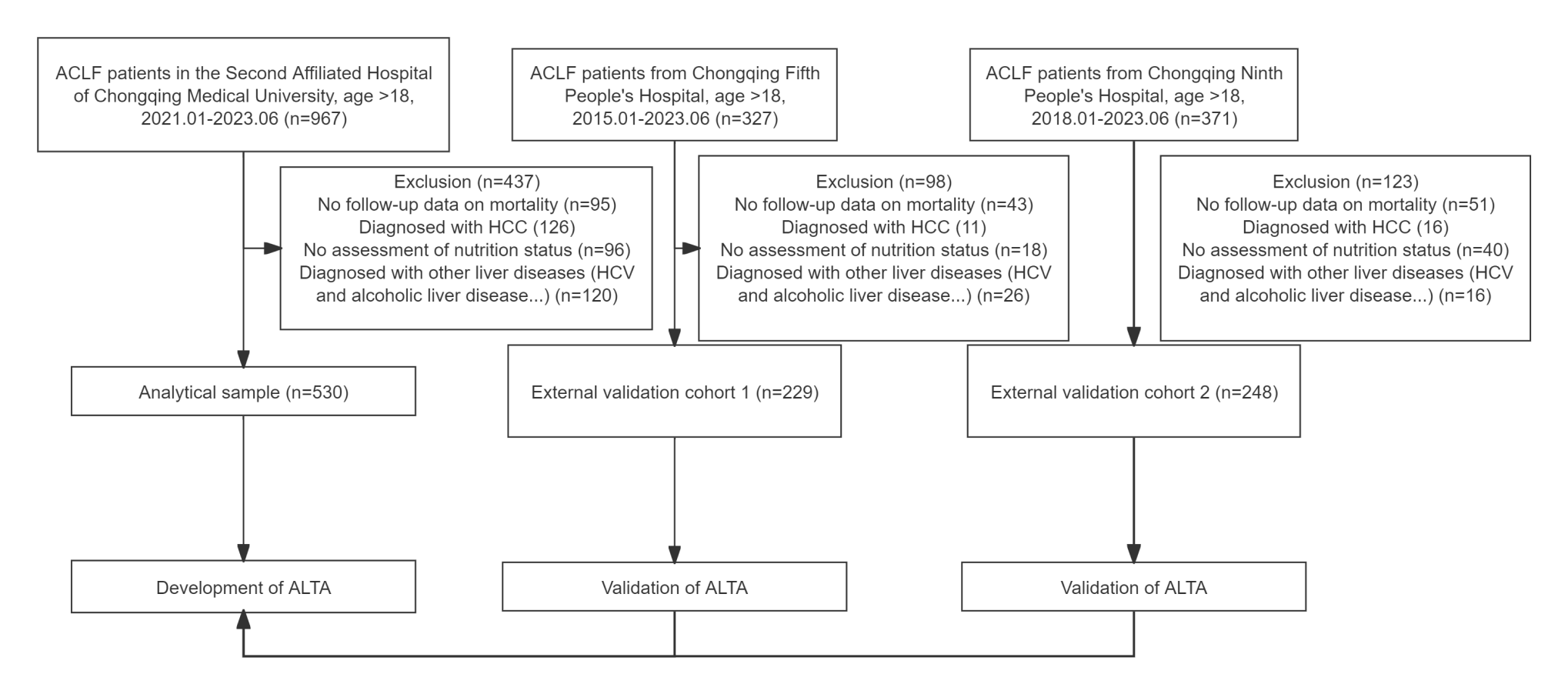


**Figure S1. Study design and flow chart of patients in this study.** ACLF: acute-on-chronic liver failure; HCC: hepatocellular carcinoma; HCV: hepatitis C virus.


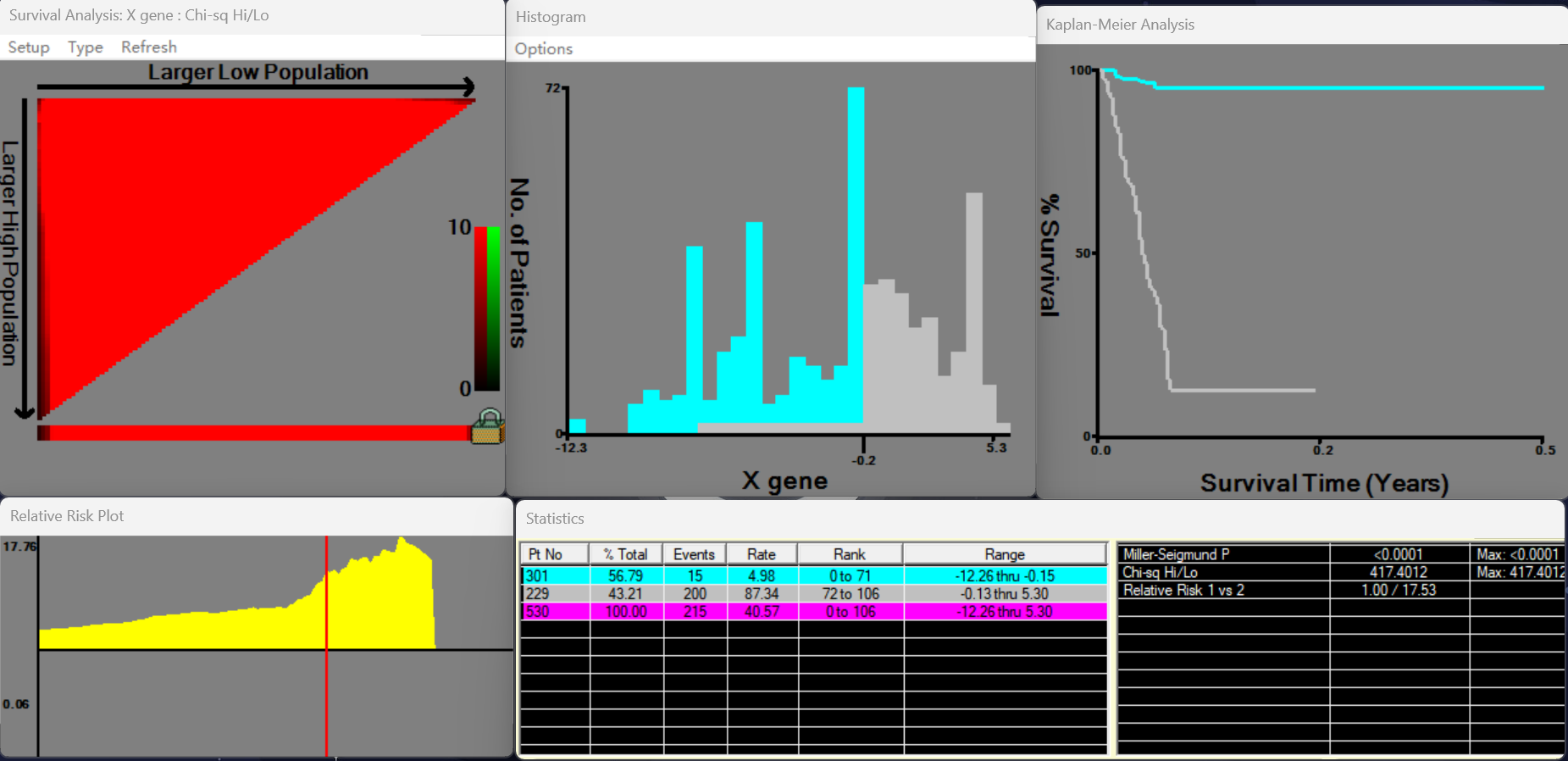


**Figure S2. The two optimal cut-off values of ALTA score selected by X-tiles**

**
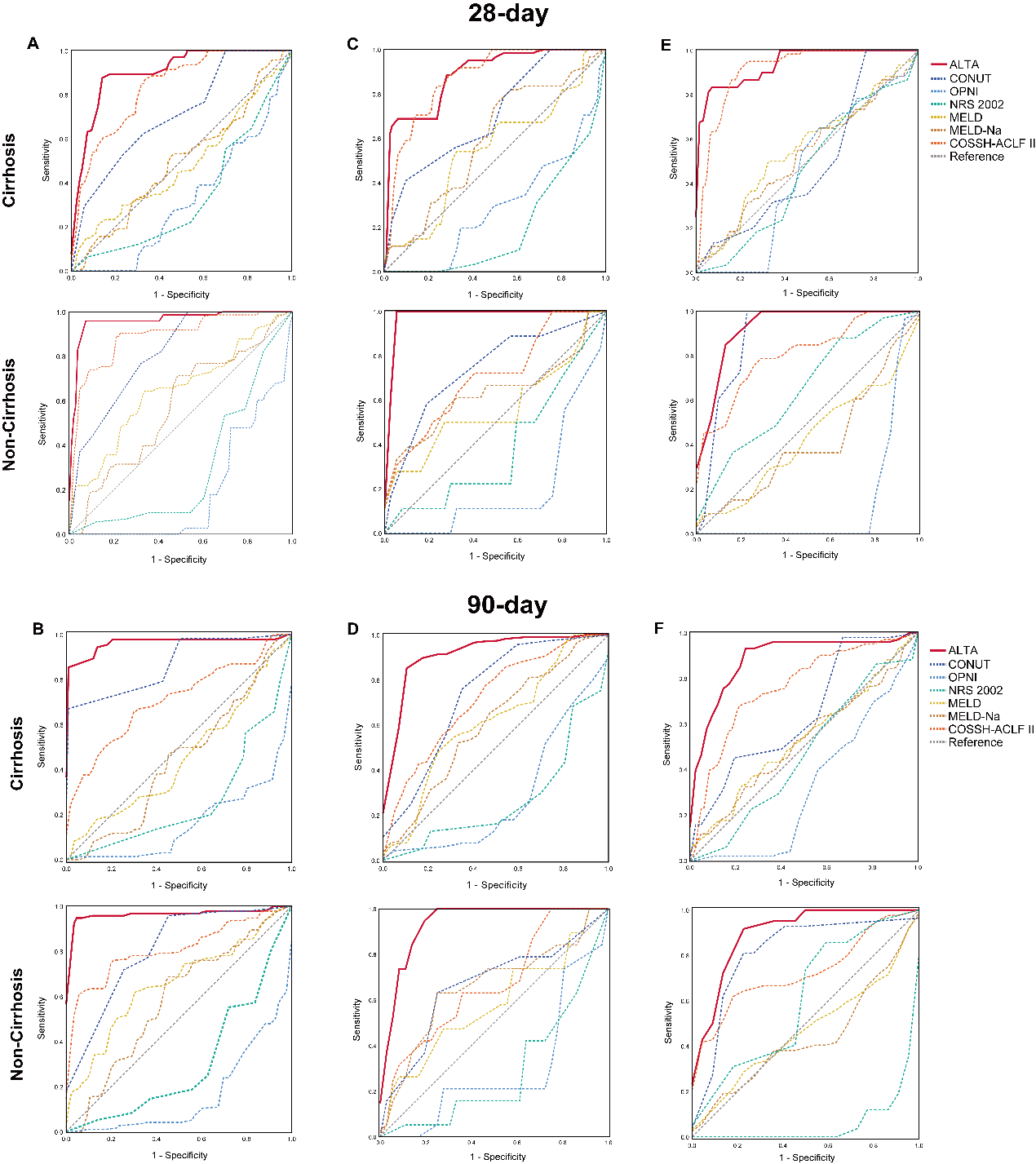
**

**Figure S3. AUROC of the ALTA score for predicting mortality of HBV-ACLF patients with and without cirrhosis in the derivation and validation cohorts.** The AUROC of the ALTA score for predicting 28-day and 90-day mortality of patients with and without cirrhosis in the derivation cohort (A) and (B), the validation cohort 1 (C) and (D), and the validation cohort 2 (E) and (F).

**Table S1. The VIF value of enrolled factors.**

| Model | Unstandardized Coefficients | | Standardized Coefficients | t | Sig. | Collinearity Statistics | |
| --- | --- | --- | --- | --- | --- | --- | --- |
|  | B | Std. Error | Beta |  |  | Tolerance | VIF |
| Constant | 0.236 | 0.302 |  | 0.719 | 0.440 |  |  |
| Age | 0.020 | 0.001 | 0.403 | 10.225 | 0.000 | 0.511 | 2.711 |
| Gender | 0.077 | 0.047 | 0.081 | 1.689 | 0.107 | 0.409 | 2.554 |
| Smoking | -0.040 | 0.053 | -0.040 | -0.611 | 0.604 | 0.228 | 4.009 |
| Drinking | -0.007 | 0.061 | -0.008 | -0.128 | 0.800 | 0.301 | 4.111 |
| HBeAg positive | 0.035 | 0.030 | 0.031 | 1.128 | 0.217 | 0.900 | 2.090 |
| Cirrhosis | -0.077 | 0.050 | -0.061 | -1.552 | 0.117 | 0.611 | 1.698 |
| Ascites | -0.001 | 0.033 | -0.001 | -0.030 | 0.900 | 0.721 | 1.411 |
| HE | -0.034 | 0.031 | -0.030 | -1.159 | 0.211 | 0.865 | 1.978 |
| Antiviral history | -0.038 | 0.036 | -0.037 | -1.029 | 0.409 | 0.599 | 2.454 |
| RBC | 0.060 | 0.019 | 0.126 | 3.010 | 0.014 | 0.511 | 1.899 |
| WBC | -0.015 | 0.004 | -0.141 | -3.111 | 0.012 | 0.409 | 2.122 |
| Lymphocyte count | -0.037 | 0.018 | -0.101 | -2.409 | 0.008 | 0.557 | 1.566 |
| Platelet | 0.002 | 0.000 | 0.139 | 3.421 | 0.015 | 0.445 | 2.122 |
| Neutrophil | 0.003 | 0.004 | 0.021 | 0.798 | 0.501 | 0.811 | 1.321 |
| Albumin | -0.014 | 0.002 | -0.150 | -4.243 | 0.000 | 0.643 | 1.609 |
| ALT | 0.000 | 0.000 | -0.163 | -4.023 | 0.000 | 0.421 | 6.787 |
| AST | 0.000 | 0.000 | -0.111 | -3.232 | 0.099 | 0.506 | 12.599 |
| TB | 0.000 | 0.000 | -0.133 | -3.011 | 0.012 | 0.467 | 2.147 |
| Serum creatinine | -8.155E-05 | 0.000 | -0.011 | -0.303 | 0.706 | 0.690 | 1.433 |
| Serum sodium | -0.004 | 0.003 | -0.047 | -1.809 | 0.079 | 0.909 | 3.432 |
| BUN | 0.000 | 0.007 | -0.002 | -0.093 | 0.778 | 0.811 | 1.990 |
| PT | 0.011 | 0.003 | 0.140 | 2.893 | 0.003 | 0.409 | 4.511 |
| PTA | -0.009 | 0.023 | -0.001 | 1.934 | 0.098 | 0.304 | 17.199 |
| INR | 0.031 | 0.005 | 0.012 | 2.023 | 1.000 | 0.395 | 12.900 |
| TC | -0.151 | 0.029 | -0.359 | -8.522 | 0.000 | 0.571 | 1.765 |

Abbreviations: VIF: variance inflation factor; HE: hepatic encephalopathy; RBC: red blood cell; WBC: white blood cell; ALT: alanine aminotransferase; TB: total bilirubin; BUN:blood urea nitrogen; PT: prothrombin time; TC: total cholesterol.

**Table S2. Univariate and multivariate logistic regression analysis of the risk factors associated with 28-day mortality in HBV-ACLF patients.**

| **Variables** | **Univariate Analysis**  **(*p* value)** | **OR (95%CI)** | **Multivariate Analysis**  **(*p* value)** | **OR (95%CI)** |
| --- | --- | --- | --- | --- |
| Age | 0.000 | 1.191 (1.156-1.226) | 0.000 | 1.206 (1.157-1.256) |
| Gender (male) | 0.643 | 0.916 (0.632-1.327) |  |  |
| Current Smoking | 0.496 | 1.130 (0.795-1.608) |  |  |
| Current Drinking | 0.475 | 0.876 (0.608-1.261) |  |  |
| HBeAg positive | 0.387 | 1.172 (0.818-1.678) |  |  |
| Cirrhosis | 0.975 | 0.995 (0.690-1.432) |  |  |
| Ascites (present) | 0.055 | 1.425 (0.992-2.407) | 0.828 | 0.919 (0.429-1.970) |
| HE (present) | 0.053 | 1.409 (0.995-1.996) | 0.374 | 1.300 (0.729-2.316) |
| Antiviral history | 0.662 | 1.082 (0.760-1.540) |  |  |
| RBC | 0.805 | 0.978 (0.819-1.168) |  |  |
| WBC | 0.162 | 0.972 (0.938-1.011) |  |  |
| Lymphocyte | 0.000 | 0.158 (0.107-0.235) | 0.009 | 0.428 (0.2265-0.809) |
| Platelet | 0.682 | 0.999 (0.996-1.002) |  |  |
| Neutrophil | 0.084 | 0.951 (0.898-1.007) | 0.598 | 0.974 (0.884-1.074) |
| Albumin | 0.015 | 0.907 (0.873-0.941) | 0.000 | 0.863 (0.806-0.923) |
| TB | 0.266 | 0.999 (0.998-1.001) |  |  |
| ALT | 0.009 | 1.000 (1.000-1.001) | 0.233 | 1.000 (1.000-1.001) |
| Serum creatinine | 0.556 | 1.001 (0.998-1.007) |  |  |
| Sodium | 0.291 | 1.011 (0.991-1.031) |  |  |
| BUN | 0.265 | 0.971 (0.923-1.022) |  |  |
| PT | 0.000 | 1.060 (1.029-1.192) | 0.562 | 0.986 (0.942-1.033) |
| TC | 0.000 | 0.150 (0.103-0.216) | 0.000 | 0.131 (0.072-0.237) |

Abbreviations: HE: hepatic encephalopathy; RBC: red blood cell; WBC: white blood cell; TB: total bilirubin; ALT: alanine aminotransferase; BUN: blood urea nitrogen; PTA: prothrombin activity; INR: international normalized ratio; PT: prothrombin time; TC: total cholesterol.

**Table S3. Baseline characteristics of HBV-ACLF patients in the external validation cohorts.**

| **Characteristics** | **Validation cohort 1 (n=229)** | **Validation cohort 2 (n=248)** |
| --- | --- | --- |
| Age, (years) | 51.0 (47.0-58.0) | 54.0 (49.0-65.0) |
| Gender, (male) | 151 (65.9) | 162 (65.3) |
| Current smoking | 83 (36.2) | 86 (34.7) |
| Current drinking | 79 (34.5) | 101 (40.7) |
| HBeAg positive | 87 (37.8) | 125 (50.4) |
| Cirrhosis | 174 (76.0) | 184 (74.2) |
| Ascites (only) | 102 (44.5) | 104 (41.9) |
| HE (only) | 99 (43.2) | 101 (40.7) |
| Acites+HE | 12 (5.2) | 19 (7.7） |
| Antiviral history |  |  |
| Present | 197 (86.0) | 201 (81.4) |
| Absent | 32 (14.0) | 45 (18.6) |
| Antiviral agents using |  |  |
| Entecavir | 96 (48.7) | 98 (48.8) |
| Tenofovir | 91 (46.2) | 92 (45.8) |
| Entecavir+Tenofovir | 10 (5.1) | 11 (15.5) |
| Laboratory examination |  |  |
| RBC (10^12^/L) | 3.3 (2.3-4.2) | 3.2 (2.6-3.9) |
| WBC (10^9^/L) | 4.4 (3.8-7.5) | 4.7 (4.0-7.8) |
| Lymphocyte (10^9^/L) | 1.4 (1.1-2.2) | 1.1 (0.7-1.5) |
| Neutrophil (10^9^/L) | 6.8 (3.9-10.0) | 6.3 (3.0-9.0) |
| Platelet (10^9^/L) | 93.0 (61.0-122.0) | 84.5 (54.3-116.5) |
| Albumin (g/L) | 31.2 (28.6-35.8) | 30.0 (26.4-32.9) |
| ALT (U/L) | 79.0 (42.0-270.0) | 66.0 (28.0-233.3) |
| TB (mg/dL) | 8.7 (5.2-13.5) | 8.5 (5.8-16.4) |
| Serum creatinine (μmol/L) | 62.7 (51.6-74.5) | 63.1 (52.9-74.1) |
| Urea (mmol/L) | 6.3 (4.0-10.3) | 7.0 (4.3-10.4) |
| Sodium (mmol/L) | 134.1 (125.6-141.7) | 134.3 (125.8-140.9) |
| PT (s) | 20.5 (18.2-24.2) | 23.2 (19.1-26.1) |
| INR | 1.6 (0.9-1.8) | 1.6 (0.9-1.8) |
| TC (mmol/L) | 2.9 (2.0-3.8) | 2.6 (1.8-3.8) |
| Nutrition status^#^ |  |  |
| Normal |  |  |
| Mild malnutrition | 46 (20.1) | 31 (12.5) |
| Moderate malnutrition | 143 (62.4) | 127 (51.2) |
| Severe malnutrition | 40 (17.5) | 93 (37.5) |

Data are presented in n (%) or median (IQR) as appropriate. ^#^The nutrition status was classified by the CONUT scoring system. Abbreviations: ALT, alanine aminotransferase; BUN, blood urea nitrogen; HE, hepatic encephalopathy; PT, prothrombin time; RBC, red blood cell; TB, total bilirubin; TC, total cholesterol; WBC, white blood cell.

**Table S4. The AUROC of ALTA for predicting 28-/90-day mortality of HBV-ACLF patients with or without cirrhosis.**

| **Model** | **ALTA** | **COSSH-ACLF II** | **MELD** | **MELD-Na** | **CONUT** | **OPNI** | **NRS2002** |
| --- | --- | --- | --- | --- | --- | --- | --- |
| **Derivation cohort** (with cirrhosis) |  |  |  |  |  |  |  |
| 28-day mortality | 0.901 | 0.834 | 0.484 | 0.497 | 0.712 | 0.319 | 0.339 |
| *p* value |  | <0.001 | <0.001 | <0.001 | <0.001 | <0.001 | <0.001 |
| 90-day mortality | 0.964 | 0.710 | 0.445 | 0.455 | 0.869 | 0.158 | 0.260 |
| *p* value |  | <0.001 | <0.001 | <0.001 | <0.001 | <0.001 | <0.001 |
| **Validation cohort 1** (with cirrhosis) |  |  |  |  |  |  |  |
| 28-day mortality | 0.887 | 0.871 | 0.549 | 0.594 | 0.705 | 0.290 | 0.200 |
| *p* value |  | 0.612 | <0.001 | <0.001 | <0.001 | <0.001 | <0.001 |
| 90-day mortality | 0.923 | 0.718 | 0.617 | 0.602 | 0.738 | 0.286 | 0.270 |
| *p* value |  | <0.001 | <0.001 | <0.001 | <0.001 | <0.001 | <0.001 |
| **Validation cohort 2** (with cirrhosis) |  |  |  |  |  |  |  |
| 28-day mortality | 0.940 | 0.912 | 0.525 | 0.551 | 0.500 | 0.448 | 0.457 |
| *p* value |  | 0.554 | <0.001 | <0.001 | <0.001 | <0.001 | <0.001 |
| 90-day mortality | 0.887 | 0.762 | 0.532 | 0.521 | 0.646 | 0.325 | 0.466 |
| *p* value |  | <0.001 | <0.001 | <0.001 | <0.001 | <0.001 | <0.001 |
| **Derivation cohort** (without cirrhosis) |  |  |  |  |  |  |  |
| 28-day mortality | 0.962 | 0.889 | 0.571 | 0.643 | 0.813 | 0.192 | 0.302 |
| *p* value |  | <0.001 | <0.001 | <0.001 | <0.001 | <0.001 | <0.001 |
| 90-day mortality | 0.969 | 0.810 | 0.581 | 0.655 | 0.842 | 0.161 | 0.304 |
| *p* value |  | <0.001 | <0.001 | <0.001 | <0.001 | <0.001 | <0.001 |
| **Validation cohort 1** (without cirrhosis) |  |  |  |  |  |  |  |
| 28-day mortality | 0.979 | 0.716 | 0.564 | 0.634 | 0.764 | 0.221 | 0.386 |
| *p* value |  | <0.001 | <0.001 | <0.001 | <0.001 | <0.001 | <0.001 |
| 90-day mortality | 0.936 | 0.667 | 0.581 | 0.684 | 0.664 | 0.298 | 0.289 |
| *p* value |  | <0.001 | <0.001 | <0.001 | <0.001 | <0.001 | <0.001 |
| **Validation cohort 2** (without cirrhosis) |  |  |  |  |  |  |  |
| 28-day mortality | 0.936 | 0.812 | 0.413 | 0.429 | 0.889 | 0.137 | 0.656 |
| *p* value |  | <0.001 | <0.001 | <0.001 | <0.001 | <0.001 | <0.001 |
| 90-day mortality | 0.906 | 0.729 | 0.436 | 0.469 | 0.801 | 0.154 | 0.612 |
| *p* value |  | <0.001 | <0.001 | <0.001 | <0.001 | <0.001 | <0.001 |
